# Cesarean Myomectomy: A Retrospective Observational Study at a Tertiary Care Center in Bihar, India

**DOI:** 10.7759/cureus.115486

**Published:** 2026-08-31

**Authors:** Poonam Lal, Deeptimayee Pathi, Swaroop R Nanda

**Affiliations:** 1 Obstetrics and Gynecology, Kurji Holy Family Hospital, Patna, IND

**Keywords:** blood loss, cesarean myomectomy, cesarean section, myoma, postoperative morbidity

## Abstract

Introduction

Fibroids during pregnancy are frequently encountered in our routine obstetric practice. Whether cesarean myomectomy is a safe and practical treatment for women with uterine myomas or if it is completely contraindicated is a topic of much discussion. Due to the apprehension of increased blood loss, there remains a dilemma in obstetricians' decisions on whether to do a cesarean myomectomy or not. Our article may help obstetricians to make an informed decision.

Method

We did a single-center, uncontrolled, retrospective observational study on 25 cases who were enrolled over two years. Data were analyzed in terms of intraoperative blood loss, need for blood transfusion, operative time, hospital stay, and postoperative morbidity.

Results

The mean age of patients in our study was 29.1 years, and the mean gestational age at delivery in our study was 37.5 weeks. In our study, the size of uterine fibroids ranged between 4 and 16 cm. The mean operative time was 68.12 minutes. The mean drop in hemoglobin between pre- and post-op was 1.1 g/dL. There was a history of blood transfusion in seven out of 25 cases. There was no significant postoperative morbidity in all the cases, except for one, and the mean hospital stay was five days. Neonatal outcome was good in 22 cases with three neonatal intensive care unit (NICU) admissions.

Conclusion

This study concluded that myomectomy is feasible with acceptable short-term outcomes with good technique in expert hands in a tertiary care center. This has the advantages of avoiding a second surgery and cost when cases are selected appropriately.

## Introduction

Uterine leiomyomas are smooth muscle tumors of the uterus that are most common in reproductive-age females [1]. The exact incidence during pregnancy is difficult to estimate. However, the literature indicates an incidence of 2-4% [2]. Fibroids complicate pregnancy by posing a higher risk of abortion, second-trimester loss, preterm birth, and malpresentation, and rates of these complications depend on the size and location of fibroids [3-5]. With the increased incidence of delayed childbearing age and the ongoing development of in vitro fertilization (IVF) techniques, there has been an increase in the number of pregnancy complications involving fibroids [6]. As we know, cesarean section is the most common surgical operation worldwide [7]. As the rate of cesarean sections has grown, obstetricians will be encountering more myomas during the procedure, and that makes knowledge on cesarean myomectomy all the more important.

The uterus vascularity grows considerably during pregnancy, and it gets 17% of the cardiac output at term, making myomectomy during pregnancy risky and perhaps necessitating hysterectomy; therefore, obstetricians are still apprehensive of doing cesarean myomectomy due to increased blood loss, the need for blood transfusion, increased postoperative morbidity, and prolonged hospital stays [8].

However, with advancements in anesthesia, adequate availability of blood products, selective devascularization techniques, and a multidisciplinary approach, obstetricians are increasingly choosing to perform myomectomy during a cesarean section, saving the patient from future morbidity due to multiple surgeries, anesthetic complications, and out-of-pocket expenses [9]. However, obstetricians should take into account various factors such as uterine contractility, anatomical localization, number and diameter of myomas, and presence of large vascular structures while performing cesarean myomectomy.

This retrospective study was carried out at a tertiary care center and aimed to analyze the perioperative maternal and neonatal outcomes in selected patients of cesarean myomectomy in terms of the amount of blood loss, the need for blood transfusion, the duration of surgery and hospital stay, and postoperative morbidity.

## Materials and methods

Study design and setting

This was a hospital-based retrospective observational study conducted in the Department of Obstetrics and Gynecology at Kurji Holy Family Hospital in Patna, Bihar, India. The study period was from January 2023 to January 2025. Data were retrospectively collected from the medical records of patients who underwent cesarean myomectomy during this period by the same team of surgeons.

The study assessed perioperative outcomes, including estimated blood loss, requirement for blood transfusion, change in hemoglobin concentration, operative duration, duration of hospital stay, postoperative morbidity, and neonatal outcome.

Study population and sampling

The study population consisted of pregnant women with uterine fibroids who underwent a cesarean section with a concomitant cesarean myomectomy during the study period. All patients who fulfilled the predefined inclusion and exclusion criteria during the study period were included in the study. The decision for myomectomy was based on the operating surgeon's assessment of the individual case, considering the size, number, location, and accessibility of the fibroid, vascularity, intraoperative bleeding, and overall maternal condition. Therefore, not all women with fibroids undergoing cesarean delivery were considered candidates for myomectomy. A total of 25 patients were included in the final analysis.

Eligibility criteria

The inclusion criteria were as follows: antenatal diagnosis of uterine myoma on ultrasonography, presence of a uterine fibroid in a patient undergoing a cesarean section for an obstetric indication, consent for cesarean myomectomy, no other surgical procedure apart from cesarean myomectomy, and no known bleeding disorder. In contrast, the exclusion criteria were the following: did not provide consent for cesarean myomectomy, had a history of a bleeding disorder, did not have blood products available during the operation, and required an additional surgical procedure apart from a cesarean myomectomy.

Data collection

Data were collected retrospectively from medical records, antenatal records, operative notes, postoperative records, and neonatal records. The variables assessed included age, parity, gestational age at delivery, obstetric risk factors, type of cesarean section, type and location of myoma, size of myoma, estimated intraoperative blood loss, preoperative and postoperative hemoglobin levels, requirement for blood transfusion, number of blood units transfused, operative duration, duration of hospital stay, postoperative morbidity, and neonatal outcome. In our study, the estimated blood loss was assessed by the operating team using visual estimation. Postoperative hemoglobin was measured 24 hours after surgery. Blood transfusion was considered based on the degree of blood loss, postoperative hemoglobin level, hemodynamic status, and clinical symptoms of anemia, rather than on hemoglobin level alone.

Surgical and hemostatic measures

Cesarean myomectomy was performed during a cesarean section in appropriately selected patients. A surgical approach to myoma resection during a cesarean myomectomy involved delivering the baby via cesarean section first, followed by careful fibroid enucleation combined with strict hemostatic techniques to control bleeding. An incision was made directly over the pseudocapsule of the fibroid, distinct from the main uterine incision if possible, and the myoma was shelled out bluntly or sharply. The myometrial bed was closed in anatomical layers using continuous or interrupted absorbable sutures (Vicryl no. 1 or 1-0) with a serosal baseball or to restore uterine integrity. Uterotonic agents, tranexamic acid, and electrocautery were used in all cases as measures to minimize intraoperative blood loss. We used carbetocin 100 IU intravenously as it is long-acting and provides sustained control over intraoperative blood loss and reduces the need for additional rescue medications. Second-line agents carboprost tromethamine (prostaglandin F2 alpha (PGF2α)) and misoprostol were kept on hand as backup treatment if carbetocin failed to stop the bleeding from the myomectomy bed.

Intravenous tranexamic acid was typically administered preoperatively, about 30 minutes before the skin incision, infused over 15-20 minutes to reduce surgical blood loss. A standard prophylactic dose is typically 1 g of tranexamic acid diluted in isotonic saline. Unilateral uterine artery ligation was performed in three patients (12%), while bilateral uterine artery ligation was performed in four patients (16%) before the enucleation of the myoma. Deciding between unilateral (one-sided) or bilateral (two-sided) uterine artery ligation during a cesarean myomectomy depends on the location, size, and vascularity of the fibroids, as well as the extent of intraoperative bleeding. Criteria for considering unilateral uterine artery ligation were a solitary fibroid confined to one lateral wall, sufficient hemostasis achieved after myomectomy, or overall minimal blood loss, whereas criteria for considering bilateral uterine artery ligation were presence of multiple fibroids or a single very large intramural/lower segment fibroid requiring extensive dissection, diffuse hypervascularity, generalized and persistent bleeding, or uterine atony after enucleation.

Outcome measures

The primary outcomes assessed were as follows: estimated intraoperative blood loss, requirement for blood transfusion, change in hemoglobin concentration, operative duration, duration of hospital stay, and postoperative maternal morbidity like wound infection, fever requiring treatment, urinary tract injury, and secondary surgical intervention. Neonatal outcome, including neonatal intensive care unit (NICU) admission, was assessed as an additional outcome.

Potential confounding factors

Potential factors that may influence perioperative blood loss and postoperative outcomes were recorded, including fibroid size and location, preoperative hemoglobin level, gestational age, obstetric risk factors, type of cesarean section, and the use of hemostatic measures such as uterine artery ligation. These variables were considered during the analysis of perioperative outcomes.

Sample size

No formal a priori sample size calculation was performed because this was a retrospective study in which all eligible cases during the predefined study period were included. The final study population consisted of 25 patients.

Statistical analysis

Data were entered into Microsoft Excel (Microsoft Corporation, Redmond, Washington, United States) and analyzed using IBM SPSS Statistics for Windows, Version 26.0 (IBM Corp., Armonk, New York, United States). Continuous variables were assessed for normality using the Shapiro-Wilk test and visual inspection of Q-Q plots. Normally distributed continuous variables were expressed as mean±standard deviation, while non-normally distributed variables were expressed as median with interquartile range. Categorical variables were summarized as frequencies and percentages. The change in hemoglobin concentration between preoperative and postoperative measurements was assessed using a paired t-test for normally distributed paired differences or the Wilcoxon signed-rank test when the normality assumption was not met. For exploratory comparison of patients who did and did not require blood transfusion, normally distributed continuous variables were compared using the independent-samples t-test and non-normally distributed continuous variables using the Mann-Whitney U test. Categorical variables were compared using Fisher's exact test when expected cell frequencies were small. Associations between continuous variables, including fibroid size, estimated blood loss, hemoglobin reduction, and operative time, were assessed using Spearman's rank correlation. 

Ethical considerations

The study was approved by the Ethics Committee of the Indian Council of Medical Research (ICMR)-Rajendra Memorial Research Institute of Medical Sciences (RMRIMS) under approval number RMRI/EC/31/2025. Informed consent for treatment and publication was obtained or waived in accordance with the institutional ethics committee requirements.

## Results

A total of 25 patients who fulfilled the eligibility criteria were included in the study. The mean age of the patients was 29.1 years, and the mean gestational age at delivery was 37.5 weeks. Most patients were between 31 and 35 years of age (40%), followed by 26-30 years (32%). Nine patients (36%) were primigravida, 12 (48%) were gravida 2, three (12%) were gravida 3, and one (4%) was gravida ≥4. Regarding gestational age, one patient (4%) delivered before <34+0 weeks, 10 (40%) between 34+0 and 36+6 weeks, and 14 (56%) ≥37+0 weeks. The demographic characteristics of the study population are summarized in Table 1.

**Table 1:** Demographic characteristics of the study population

Variable	Category	Number of cases (n=25)	Percentage (%)
Age (years)	20-25	3	12
	26-30	8	32
	31-35	10	40
	36-40	4	16
Gravidity	Primigravida	9	36
	Gravida 2	12	48
	Gravida 3	3	12
	Gravida ≥4	1	4
Gestational age at delivery (weeks)	<34+0	1	4
	34+0-36+6	10	40
	≥37+0	14	56

Among the obstetric risk factors, a previous lower segment cesarean section was the most common, occurring in eight patients (32%). Placenta previa was present in one patient (4%), prelabor rupture of membranes (PROM)/preterm prelabor rupture of membranes (PPROM) in three (12%), obstetric cholestasis in two (8%), and twin pregnancy in one (4%). Four patients (16%) had other risk factors, while six (24%) had no identifiable obstetric risk factor. Twenty-one patients (84%) underwent elective cesarean sections, and four (16%) underwent emergency cesarean sections. The obstetric characteristics of the study population are presented in Table 2.

**Table 2:** Obstetric characteristics of the study population

Variable	Category	Number of cases (n=25)	Percentage (%)
Obstetric risk factors	Previous lower segment cesarean section	8	32
	Placenta previa	1	4
	Prelabor rupture of membranes/preterm prelabor rupture of membranes	3	12
	Obstetric cholestasis	2	8
	Twin pregnancy	1	4
	Others	4	16
	None	6	24
Type of cesarean section	Elective	21	84
	Emergency	4	16

Intramural fibroids were the most common type, occurring in 15 patients (60%), followed by subserosal fibroids in five (20%), cervical fibroids in four (16%), and submucosal fibroids in one (4%). Among these fibroids, seven were located in the lower uterine segment, six were located in the anterior wall, five were located in the posterior wall, one was located at the cornu, two were located at the uterine angle, and four of them were cervical fibroids. Regarding size, 13 fibroids (52%) measured 4-8 cm, six (24%) measured 8-12 cm, and six (24%) measured >12 cm. The largest fibroid measured 16 cm. The distribution of fibroid type, location, and size is summarized in Table 3.

**Table 3:** Characteristics of uterine fibroids according to type, location, and size

Variable	Category	Number of cases (n=25)	Percentage (%)
Type of fibroid	Intramural	15	60
	Subserosal	5	20
	Submucosal	1	4
	Cervical	4	16
Location of fibroids	Cornual	1	-
	Uterine angle	2	-
	Lower uterine segment	7	-
	Anterior wall	6	-
	Posterior wall	5	-
	Cervical	4	-
Fibroid size (cm)	4-8	13	52
	8-12	6	24
	>12	6	24

The estimated intraoperative blood loss ranged from 500 to >1000 mL. Blood loss was 500-699 mL in 20 patients (80%), 700-1000 mL in two patients (8%), and >1000 mL in three patients (12%). The mean preoperative hemoglobin level was 11.208 g/dL, the mean postoperative hemoglobin level was 10.095 g/dL, and the mean fall in hemoglobin was 1.113 g/dL. Perioperative blood loss and hemoglobin changes are summarized in Table 4.

**Table 4:** Perioperative blood loss and changes in hemoglobin concentration

Variable	Category/measure	Number of cases (n=25)	Percentage (%)
Estimated blood loss (mL)	500-699	20	80
	700-1000	2	8
	>1000	3	12
Hemoglobin (g/dL)	Mean preoperative hemoglobin	11.208	-
	Mean postoperative hemoglobin	10.095	-
	Mean fall in hemoglobin	1.113	-

Blood transfusion was required in seven patients (28%), whereas 18 patients (72%) did not require transfusion. Among the seven patients who received transfusions, four received one unit, two received two units, and one received three units. The patient requiring three units had a 16-cm intramural lower uterine segment fibroid, with a preoperative hemoglobin level of 8.5 g/dL. She underwent emergency cesarean section with approximately 1300 mL blood loss. Blood transfusion requirements are summarized in Table 5.

**Table 5:** Blood transfusion requirements among patients undergoing cesarean myomectomy

Variable	Category	Number of cases (n=25)	Percentage (%)
Blood transfusion	No	18	72
	Yes	7	28
Units transfused among transfused patients (n=7)	1 unit	4	57.1
	2 units	2	28.6
	3 units	1	14.3

The mean operative time was 68.12 minutes. Four patients (16%) had an operative time of <1 hour, 15 (60%) had an operative time of 1-1.5 hours, five (20%) had an operative time of 1.5-2 hours, and one patient (4%) had an operative time >2 hours. The mean hospital stay was approximately five days. Twelve patients (48%) were discharged after five days, five (20%) after six days, and seven (28%) after seven days, and one patient (4%) required >7 days of hospitalization. The distribution of operative time and hospital stay is presented in Table 6.

**Table 6:** Operative duration and duration of hospital stay

Variable	Category	Number of cases (n=25)	Percentage (%)
Operative time	<1 hour	4	16
	1-1.5 hours	15	60
	1.5-2 hours	5	20
	>2 hours	1	4
Hospital stay	5 days	12	48
	6 days	5	20
	7 days	7	28
	>7 days	1	4

Significant postoperative morbidity was recorded in one patient (4%), while 24 patients (96%) had no significant postoperative morbidity. The patient with morbidity developed a mild postoperative infection. No patient developed intestinal obstruction, including those undergoing posterior wall myomectomy. NICU admission was required in three neonates (12%), with prematurity being the reported reason for admission. Maternal postoperative morbidity and neonatal outcomes are summarized in Table 7.

**Table 7:** Maternal postoperative morbidity and neonatal outcomes

Variable	Category	Number of cases (n=25)	Percentage (%)
Significant postoperative morbidity	Yes	1	4
	No	24	96
Neonatal intensive care unit admission	Yes	3	12
	No	22	88

Uterotonics, tranexamic acid, and electrocautery were used in all 25 cases (100%) as measures to reduce intraoperative blood loss. Unilateral uterine artery ligation was performed in three patients (12%), while bilateral uterine artery ligation was performed in four patients (16%) before enucleation. The intraoperative measures used to reduce blood loss are summarized in Table 8.

**Table 8:** Intraoperative measures used to reduce blood loss during cesarean myomectomy

Measure	Number of cases (n=25)	Percentage (%)
Uterotonics	25	100
Tranexamic acid	25	100
Electrocautery	25	100
Unilateral uterine artery ligation	3	12
Bilateral uterine artery ligation	4	16

Figure 1 shows a large intramural fibroid (16 cm in size) during a cesarean myomectomy.

**Figure 1:**
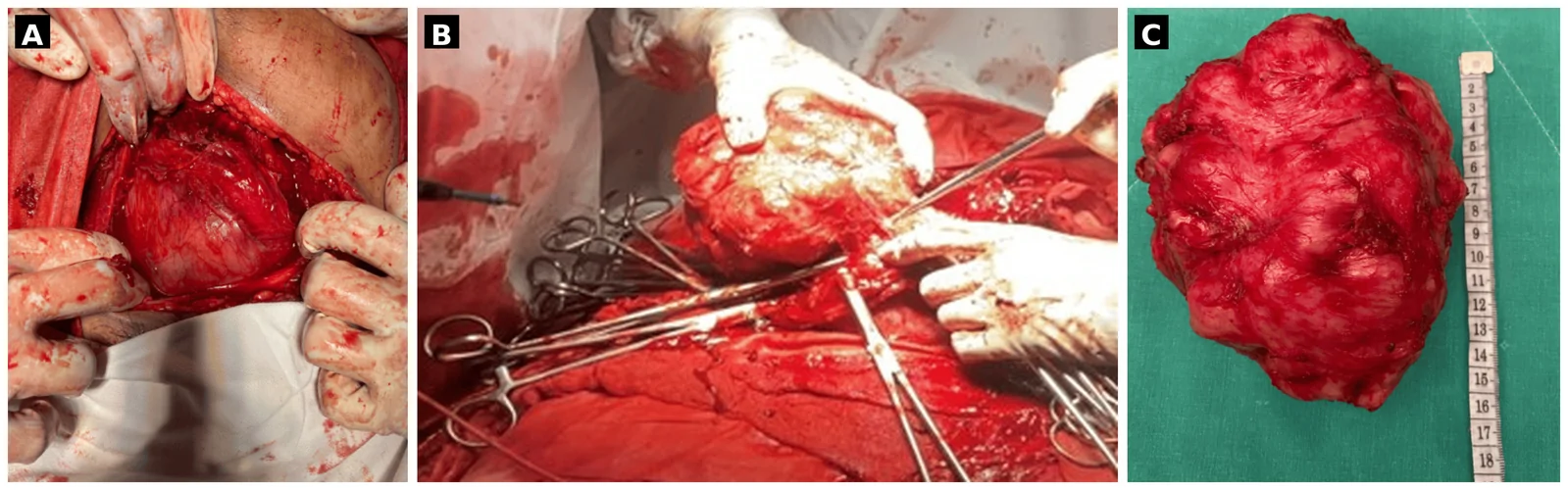
Large intramural fibroid during cesarean myomectomy (A) Anterior wall intramural fibroid. (B) Use of electrocautery and clamps for hemostasis. (C) Intramural fibroid (16 cm).

Figure 2 shows myomas at technically difficult locations during cesarean myomectomy.

**Figure 2:**
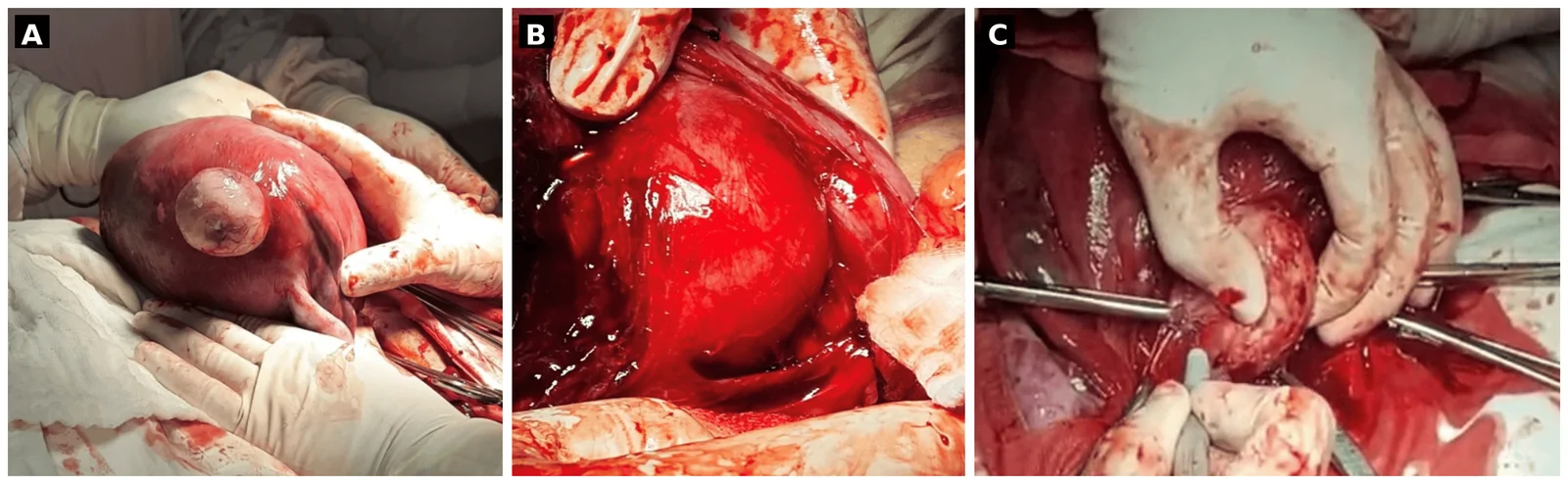
Myomas at technically difficult locations during cesarean myomectomy (A) Myoma at the right cornu of the uterus. (B) Myoma at the left angle of the uterine incision. (C) Myoma at the right angle of the uterine incision.

Figure 3 illustrates a large posterior wall submucosal myoma seen during the closure of the uterine incision after the delivery of the fetus.

**Figure 3:**
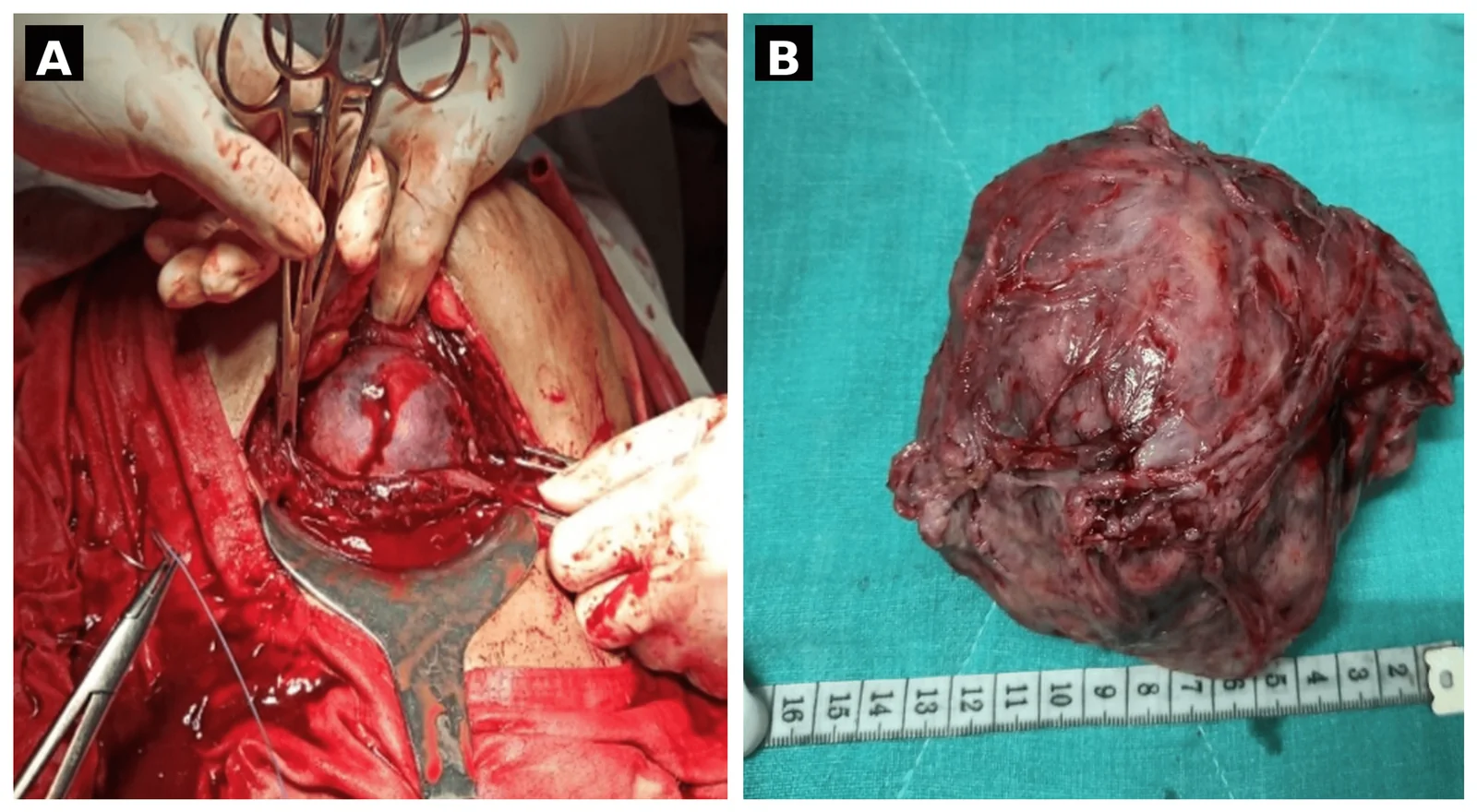
Submucosal myoma in the posterior wall of the uterus (A) Submucosal myoma in the posterior wall seen through lower segment cesarean section incision. (B) Myoma (13 cm).

Figure 4 demonstrates an intramural fibroid (10 cm in size) at the upper border of the uterine incision.

**Figure 4:**
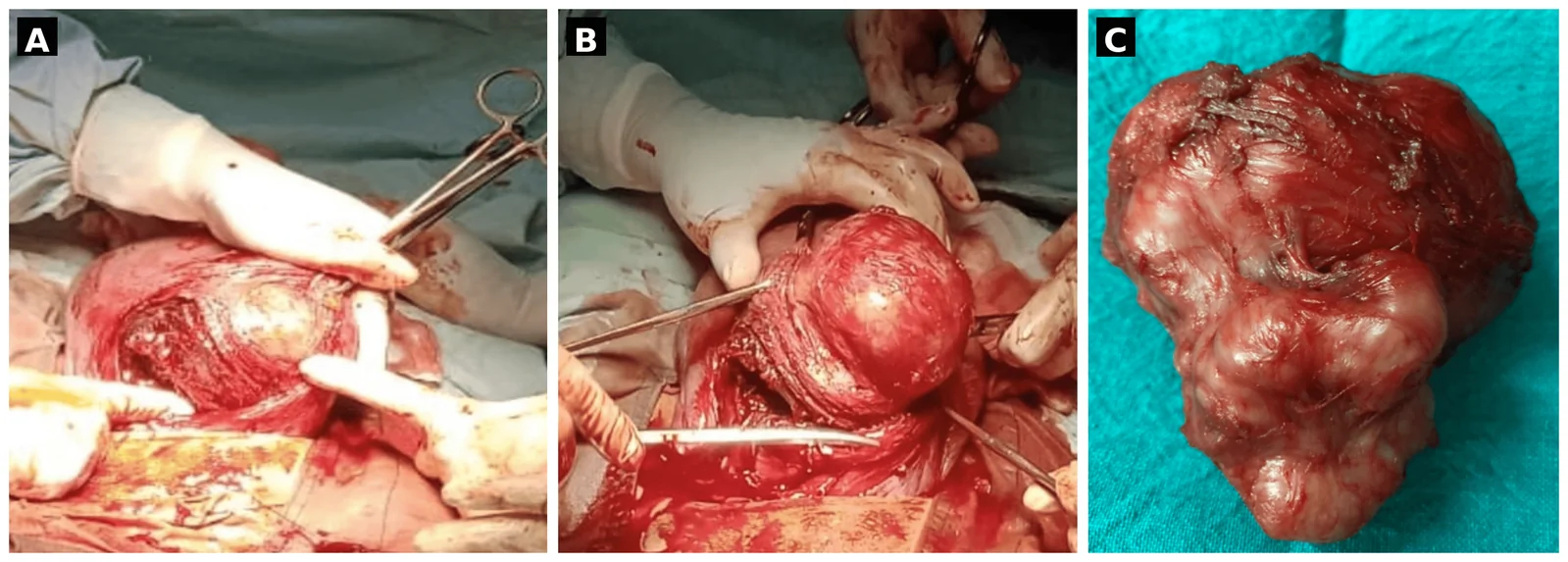
Intramural fibroid (10 cm) at the upper border of the uterine incision (A) A 10 cm intramural fibroid at the upper border of the uterine incision. (B) Enucleation of the fibroid. (C) Retrieved specimen.

Figure 5 demonstrates the dissection of a posterior wall subserosal myoma with minimal blood loss.

**Figure 5:**
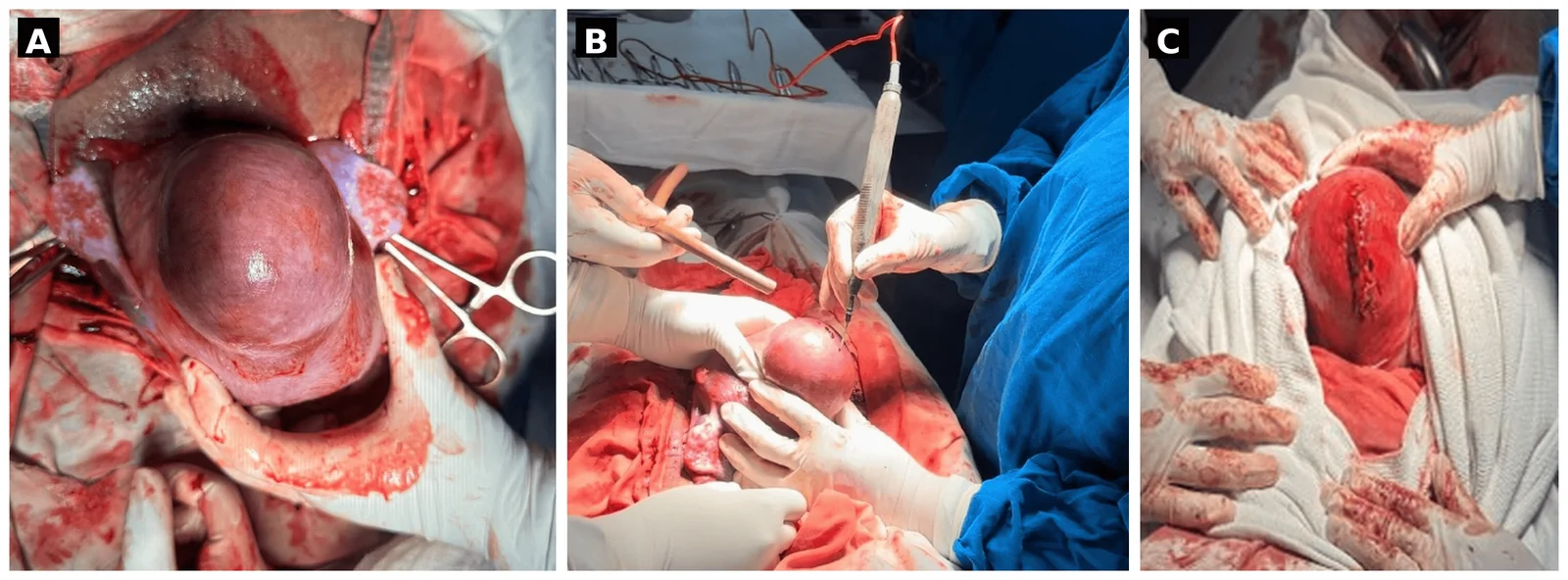
Enucleation of a posterior wall subserous myoma (A) Posterior wall myoma. (B) Use of electrocautery in dissection. (C) Final picture after the removal of the myoma.

## Discussion

Apprehension for cesarean myomectomy is attributed to perioperative blood loss, prolonged surgery, increased postoperative morbidity, and prolonged hospital stays [10]. This apprehension is especially more for intramural myomas and myomas at difficult locations in comparison with pedunculated and subserous fibroids. Moreover, the advantages of doing cesarean myomectomies are easy enucleation, avoidance of later surgery and ultrasound, symptom relief, and reduced complications in the puerperium and the next pregnancy [10].

Proper selection of patients is mandatory before doing myomectomy. With greater awareness of healthcare, patients are worried about fibroids, undergo unnecessary ultrasounds, and demand myoma removal during a cesarean section. Sometimes, obstetricians may encounter larger intramural myomas in the lower segment, causing difficulty in the closure of the uterine incision, necessitating its removal. Obstetricians should be aware of its indications and techniques to perform a safe cesarean myomectomy.

Common indications discussed in various literature are symptomatic pain, the patient's desire, a single myoma more than 5 cm, pedunculated and subserous myomas, and intramural myomas in the lower uterine segment [10]. In a meta-analysis and systematic review by Goyal et al., including 17 studies and 6545 patients, they reported no difference in the incidence of hemorrhage between cesarean myomectomy and cesarean section but a statistically significant increase in the odds of blood transfusion with cesarean myomectomy. The mean myoma size was 5-10 cm; there was an increase in operative time (approximately 15 minutes) with increased odds of hospital stay for cesarean myomectomy patients [11]. The mean age of cases in our study was 29.1 years; most of the cases (48%) were second gravida. The average gestational age at the time of cesarean section was 37.5 weeks in our study, which is similar to other studies [8-12]. Only three patients underwent an emergency cesarean section at 34 weeks in view of antepartum hemorrhage or fetal distress. The range of myomas in our study was from 4 cm to 16 cm.

The main indication for myomectomy in seven out of the 25 cases was the location of the myoma at the lower uterine segment, which necessitated its removal to properly close the uterine incision. However, one case of a 13 cm submucosal fibroid was discovered at the time of lower segment cesarean section, which was diagnosed as intramural on antenatal ultrasonography. One patient with an intramural posterior wall fibroid of 7 cm insisted on a cesarean myomectomy at the time of the cesarean section. In two cases with small-sized myomas, one of 4 cm (near the uterine artery) and one of 5 cm (right cornu), respectively, patients were so obsessed with the fibroids that they correlated every symptom with the fibroids and underwent several ultrasonographies over a period of two years. They requested and insisted on removing the myoma, and we performed it, respecting the patient's wish to prevent future surgery and out-of-pocket expenditure.

We operated on seven cases of intramural fibroids at the lower uterine segment, the largest being 16 cm, and in our experience, we found that intramural myomas are better dissected in the space between the subendometrium and myometrium due to more defined planes in pregnancy with minimum blood loss, especially with the use of electrocautery. Moreover, closure of the incision could be done by simply approximating the edges of the uterine incision. A study by Garg and Bansal also found that enucleation of the fibroid was easier in pregnancy, as the postpartum uterus is better adapted physiologically to control bleeding than in any other phase of a woman's lifetime [6].

In view of the patient's request and after taking informed consent, we removed one cornual fibroid of 5 cm and four cases of posterior wall fibroids, which are traditionally avoided due to the anticipation of heavy bleeding and risk of postoperative adhesion formation, respectively. In our study, the mean blood loss was 656 mL; only seven out of 25 cases (28%) required blood transfusion. Out of these seven cases, only one case required three units of blood, one required two units, and the remaining five cases required one unit each. The case that required three units of blood was the case of a 16 cm intramural fibroid at the lower uterine segment. Her blood loss was approximately 1300 mL attributed to its large size. Moreover, her pre-op hemoglobin was 8.5 g/dL. There was no need for blood transfusion in the remaining 18 cases that could be attributed to intraoperative measures taken to prevent hemostasis.

Many authors advocated specific hemostatic measures like intraoperative tranexamic acid, uterotonics, and the use of cautery [13,14]. In all 25 cases, we used intraoperative tranexamic acid, uterotonic agents, and electrocoagulation for dissection. Bilateral uterine artery ligation was done for four cases of larger myomas in anticipation of massive hemorrhage. Unilateral uterine artery ligation was done in three cases, especially where the myoma was located at the angle of incision very near the uterine artery. A study done by Singh et al. also emphasized the role of stepwise devascularization in controlling blood loss [13].

A retrospective case-control study by Kaymak et al. comparing 40 women with fibroids who underwent cesarean myomectomy with 80 women with fibroids forming the control group who underwent cesarean section alone reported no significant difference in the incidence of hemorrhage between the two groups (12.5% and 11.3%, respectively) [15].

The mean operative time in our study was 68.12 minutes, which is only 23 minutes more than the routine operative time for a cesarean section (45 minutes). The mean hospital stay was five days, which is similar to the usual hospital stay protocol for cesarean sections at our center. In our study, only one patient who was anemic developed a mild infection and stayed for seven days. There was no postoperative morbidity in the rest of the 24 cases (96%). Neonatal outcome in terms of Apgar score was good in 22 cases, and three NICU admissions were required in view of prematurity.

Ramya et al., in their retrospective study of 20 cases, also concluded no postoperative morbidity with a mean hospital duration of five days [14]. The same was reemphasized by the meta-analysis done by Goyal et al. [11]. Parveen et al. studied eight patients with cesarean myomectomy and reported that cesarean myomectomy added 20 extra minutes to the cesarean section and the hospital stay was the same as that of the post-cesarean section, with good neonatal outcomes in all the cases [16]. Their results were similar to our study.

Histopathology reports of all 25 cases were reviewed, and no malignant changes were seen in any of the cases. Many case reports and series in the available literature also advocated the safety of cesarean myomectomy in terms of perioperative blood loss, the need for blood transfusion, operative time, and hospital stay. Table 9 depicts the key observations among various studies done on cesarean myomectomy [11,14,17-19].

**Table 9:** Relevant studies on CM by different researchers CM: cesarean myomectomy; CS: cesarean section

Author	Study design	Sample size	Key findings
Goyal et al. [11]	Review and meta-analysis	6545 cases (17 studies)	CM was associated with a clinically insignificant increase in operative time, blood loss, and hospital stay
Gürsoy et al. [17]	Retrospective study	54	No significant difference in postoperative fever, preoperative hemoglobin, or change in hemoglobin levels following CM
Ramya et al. [14]	Retrospective study	20	Most patients were discharged on the fifth postoperative day, with no incidence of postpartum morbidity
Zhao et al. [18]	Retrospective cohort study	2344	CM was found to be safe and feasible when performed by experienced obstetricians. Special attention should be given to leiomyomas ≥5 cm
Jha et al. [19]	Retrospective study	12	CM during CS can avoid a second surgery for uterine fibroids, with an acceptable risk of blood transfusion

There are certain limitations to our study. The retrospective methodology, absence of a control group, strong selection bias, single-center design, and small sample size limit the generalizability of the results. This study also lacks long-term follow-up with these patients, which would emphasize the influence on future pregnancies.

## Conclusions

Performing a cesarean myomectomy can be safe if done by an experienced surgeon. A cesarean myomectomy removes the need for two separate procedures and related treatment expenditures, which is very important from the patient's perspective. An intramural fibroid obstructing the lower uterine segment or an accessible serous and pedunculated fibroid in a symptomatic patient can be safely removed at the time of cesarean section. Measures to minimize blood loss significantly decrease the need for blood transfusion, postoperative morbidity, and chances of hysterectomy.

At present, cesarean myomectomy remains an individualized decision rather than a universally recommended routine procedure. Our study suggests that cesarean myomectomy can be safely performed in appropriately selected patients and experienced hands; however, larger prospective studies and randomized controlled trials are needed before it can be considered a routine procedure. Our findings should therefore be interpreted in the context of careful patient selection and the resources and expertise available at the treating institution.
